# Genomic vulnerability and socio‐economic threats under climate change in an African rainforest bird

**DOI:** 10.1111/eva.13193

**Published:** 2021-01-28

**Authors:** Thomas B. Smith, Trevon L. Fuller, Ying Zhen, Virginia Zaunbrecher, Henri A. Thomassen, Kevin Njabo, Nicola M. Anthony, Mary K. Gonder, Wolfgang Buermann, Brenda Larison, Kristen Ruegg, Ryan J. Harrigan

**Affiliations:** ^1^ Center for Tropical Research Institute of the Environment & Sustainability University of California Los Angeles Los Angeles CA USA; ^2^ Department of Ecology and Evolutionary Biology University of California Los Angeles Los Angeles CA USA; ^3^ Zhejiang Provincial Laboratory of Life Sciences and Biomedicine Key Laboratory of Structural Biology of Zhejiang Province School of Life Sciences Westlake University Hangzhou China; ^4^ Institute of Biology Westlake Institute for Advanced Study Hangzhou China; ^5^ University of Tübingen Tübingen Germany; ^6^ Department of Biological Sciences University of New Orleans New Orleans LA USA; ^7^ Department of Biology Drexel University Philadelphia PA USA; ^8^ Institute of Geography Augsburg University Augsburg Germany; ^9^ Department of Biology Colorado State University Fort Collins CO USA

**Keywords:** Central Africa, climate change, conservation biology, evolutionary genomics, genomic vulnerability

## Abstract

Preserving biodiversity under rapidly changing climate conditions is challenging. One approach for estimating impacts and their magnitude is to model current relationships between genomic and environmental data and then to forecast those relationships under future climate scenarios. In this way, understanding future genomic and environmental relationships can help guide management decisions, such as where to establish new protected areas where populations might be buffered from high temperatures or major changes in rainfall. However, climate warming is only one of many anthropogenic threats one must consider in rapidly developing parts of the world. In Central Africa, deforestation, mining, and infrastructure development are accelerating population declines of rainforest species. Here we investigate multiple anthropogenic threats in a Central African rainforest songbird, the little greenbul (*Andropadus virens*). We examine current climate and genomic variation in order to explore the association between genome and environment under future climate conditions. Specifically, we estimate *Genomic Vulnerability*, defined as the mismatch between current and predicted future genomic variation based on genotype–environment relationships modeled across contemporary populations. We do so while considering other anthropogenic impacts. We find that coastal and central Cameroon populations will require the greatest shifts in adaptive genomic variation, because both climate and land use in these areas are predicted to change dramatically. In contrast, in the more northern forest–savanna ecotones, genomic shifts required to keep pace with climate will be more moderate, and other anthropogenic impacts are expected to be comparatively low in magnitude. While an analysis of diverse taxa will be necessary for making comprehensive conservation decisions, the species‐specific results presented illustrate how evolutionary genomics and other anthropogenic threats may be mapped and used to inform mitigation efforts. To this end, we present an integrated conceptual model demonstrating how the approach for a single species can be expanded to many taxonomically diverse species.

## 1 INTRODUCTION

To avoid extinction under future climate change, species will either need to move to more suitable areas, or respond in situ, through plastic and/or adaptive mechanisms (Gienapp et al., 2007). Because mutations rates are generally too low to keep up with rapid environmental changes, sufficient standing genetic variation in relevant traits must be present for populations to be able to adapt (Carroll et al., 2014; Smith et al., 2014). Mitigating the threat of extinction posed by climate change will almost certainly require some level of understanding of the evolutionary processes at work. However, evolutionary considerations are seldom incorporated into conservation planning (Smith et al., 2014). Historically, conservation decisions have placed greater emphasis on ecological features, and the *patterns* of biodiversity, such as species richness and levels of regional endemism. While the evolutionary *processes* that maintain and generate biodiversity have long been recognized as fundamentally important in conservation planning (Pressey et al., 2003; Smith, 1993), accelerating anthropogenic change is increasing the importance of having an evolutionary perspective. However, omitting some aspects is understandable, as detailed information on evolutionary and genetic processes can be challenging to acquire. In contrast, information on the occurrence and abundance of species has long been available and used to guide conservation plans. While these standard metrics have been fundamentally important for inclusion in conservation planning, they alone may no longer be sufficient, particularly for regions experiencing rapid change. However, combining them with evolutionary data based on genomic variation across populations has great utility for preserving species and may offer critical bet‐hedging approaches for buffering populations against declines (Carroll et al., 2014; Hendry et al., 2011; Smith et al., 2014). In other words, maximizing genetic variation can enhance the potential for natural selection to act in ways that allow populations to ultimately adapt and persist.

Of course, the threat posed by future climate change is only one of many, on a planet where two‐thirds of the terrestrial land area is devoted to anthropogenic demands such as agriculture, infrastructure, and urbanization (Millennium Ecosystem Assessment, 2005). To maximize the success of conservation efforts to be successful, it is important to consider all possible potential current and future threats. This is particularly important for Africa's Congo Basin, where there is ongoing and intense development and extraction of natural resources (Fuller et al., 2019). Often referred to as ground zero for climate change, the Congo Basin's challenges are predicted to be enormous (Dargie et al., 2017). Africa harbors one out of every five bird and mammal species on the planet, sequesters an estimated 90Gt of carbon, and will be home to four out of every ten humans before the end of the century. Some estimates suggest Africa may lose 30% of its species if the global mean temperature increases by 1.5°C over preindustrial levels, a threshold likely to be reached soon given current trends in CO_2_ emissions (CSC, 2013; IPCC, 2018). Mitigating the effects of major climatic shifts along with impacts from natural resource exploitation, rapid human population growth, and urbanization presents enormous challenges.

Here we take an integrated approach for exploring the complex pressures that climate change and human development impose on species, in order to create a framework that explicitly considers adaptive variation under both contemporary and future anthropogenic conditions. To demonstrate the utility of this framework, we examine these conservation considerations for a single species, the common rainforest bird the little greenbul (*Andropadus virens*). The little greenbul is a species that is abundant in many types of habitats, including mature and secondary forest, agricultural lands, and the ecotone between rainforests and savanna. The species has been the subject of numerous evolutionary genetic and ecological studies for more than 20 years (Smith et al., 1997, 2001, 2005, 2008, 2013; Smith & Grether, 2008; Zhen et al., 2017).

The goals of this paper are three‐fold: (1) to explore how the current patterns of intraspecific genomic variation and their environmental correlates can be used to identify priority areas for conservation under future climate change, (2) to integrate this information with projected anthropogenic impacts from natural resource extraction (e.g., logging and mining), infrastructure, and plans for large‐scale agriculture (Edwards et al., 2014; Gillet et al., 2016; Mahmoud et al., 2017; Sloan et al., 2017) for a single species to illustrate the approach, and (3) building off these goals, to develop a comprehensive approach and road map that is applicable for multiple species and communities. In other words, leveraging past research on this species will ultimately allow us to include additional data from a diverse set of other species (e.g., mammals, plants, and insects) as it becomes available and ultimately provide a more comprehensive approach for conservation planning.

## 2 METHODS

We mapped environmentally associated genomic variation in an abundant rainforest bird under current and projected future climate conditions and assessed how these overlap with socio‐economic threats, patterns of species richness and endemism, and existing protected areas.

### 2.1 Assessing socio‐economic threats

We examined a number of threats from socio‐economic activities, including extractive industries such as logging and mining, large‐scale agriculture, human population size, and major infrastructure projects. There are increasing concerns across Central Africa that the extraction of minerals including gold, bauxite, cobalt, diamonds, iron, and rare earth elements could threaten biodiversity. A salient example is the Mbalam iron ore concession located in close proximity to the Dja Biosphere Reserve (Edwards et al., 2014). We obtained maps of active mining concessions from Cameroon's Ministry of Mine and Technological Development and the World Resources Institute (WRI et al., 2016; Figure S1).

We also assembled maps of logging concessions from an atlas developed by Cameroon's Ministry of Forestry and Wildlife and the World Resources Institute (WRI, 2012; WRI et al., 2016). The concessions included active cutting areas where timber harvest is ongoing and production forests where timber harvest is permitted by law (Figure S2). Finally, we attempted to quantify current and future large‐scale agriculture, human population densities, and major infrastructure (see Figures S3–S5, Table S1).

### 2.2 Endemism and species richness

Species data were obtained from IUCN, NatureServe, and BirdLife International databases on amphibians, birds, freshwater fish, mammals, plants, and reptiles (Hoekstra et al., 2010; Jenkins et al., 2013; Table S2). Because our study focused on terrestrial species only, coastal and pelagic species were not included in our analyses. Downloaded data consisted of GIS feature layers for each species, which were clipped to the extent of Cameroon using ArcGIS 10.1 (ESRI). Richness was calculated as the cumulative number of species per site at the 1 km^2^ scale. We defined endemism as only those species found within the borders of Cameroon.

### 2.3 Protected areas

While there is a variety of land management practices for preserving biodiversity, ranging from easements to biodiversity‐friendly agriculture, the main approach in Central Africa is through the establishment of protected areas (Kelly & Gupta, 2016). In Cameroon, protected areas presently cover approximately 10.6% of the country's terrestrial surface area (Takem et al., 2010). We compiled a GIS map of these protected areas by querying the World Database on Protected Areas (WDPA) (UNEP‐WCMC & IUCN, 2017). We filtered the WDPA data set for protected areas for which the exact geographic boundaries were available.

### 2.4 Understanding the current genome–environment relationships

**FIGURE 1:**
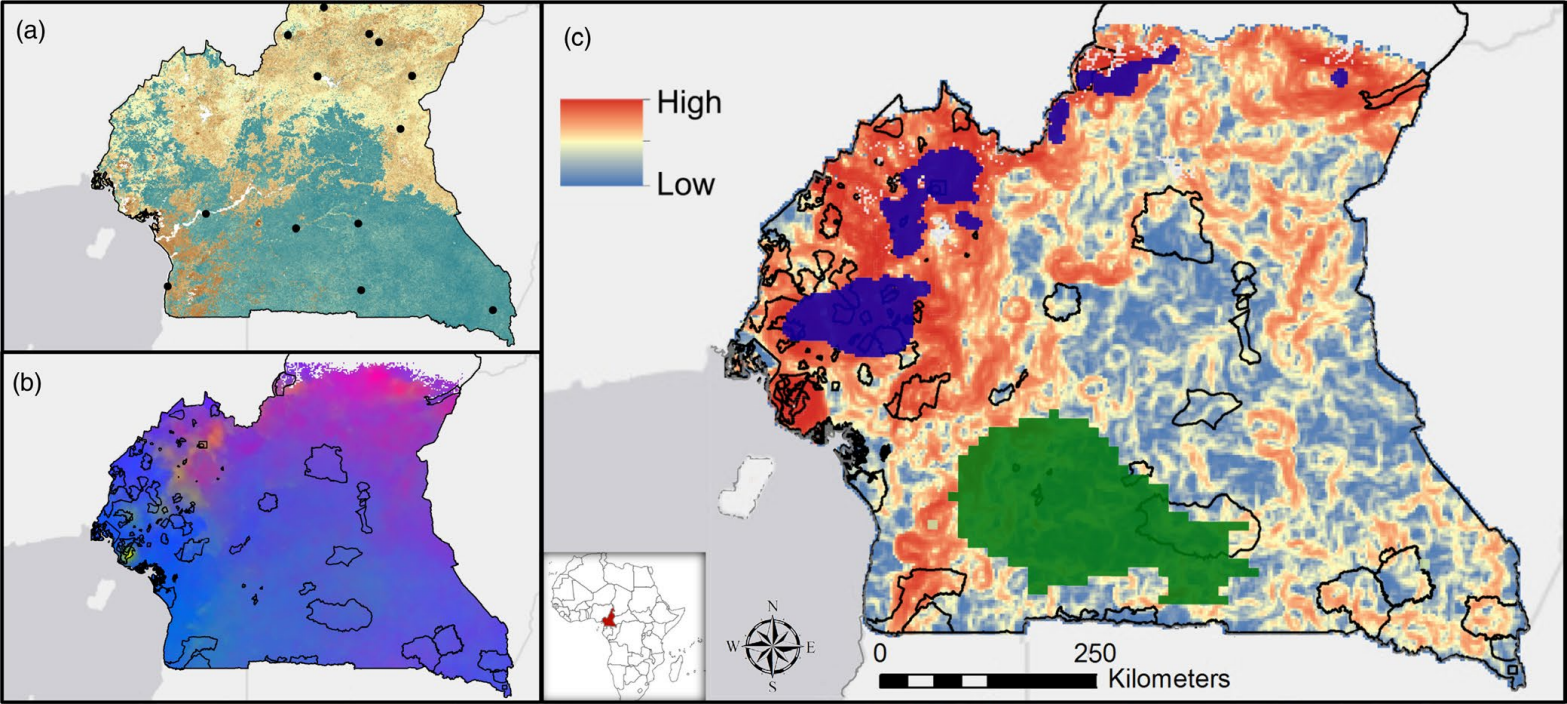
Genomic variation and turnover of the little greenbul across its range (a) with sample locations in Cameroon indicated and forest cover represented as background. Variation in the genome of a species can be visualized by color (b), where greater differences in colors represent greater adaptive genomic variation between populations across environments ((a) and (b) modified from Zhen et al., 2017). These differences are further quantified and represented in (c), with higher (red) or lower (blue) adaptive turnover across regions. High turnover areas vary in their correspondence to species richness (green polygons) or endemism (blue polygons) (see text for details). Current protected areas are represented by black‐outlined polygons in (b) and (c)

Previous research on the little greenbul found genetic variation across rainforest, montane, and ecotone habitats (Smith et al., 1997, 2005). In this study, we use previous genetic analyses by Zhen et al. (2017) and samples collected from across habitats (Figure 1a). The little greenbul's evolutionary history has been shaped by range expansion since the Last Glacial Maximum (LGM, 20,000 before present (Cahen & Snelling, 1984; Clark et al., 2009; Dalibard et al., 2014; Dupont & Weinelt, 1996; Eno Belinga, 1984; Maley, 2001; Wright, 1985) (Figure S6). Since that time, greenbuls have expanded their distribution across Cameroon. Greenbul allele frequencies differ in rainforest, ecotone, and montane sites (Zhen et al., 2017), which likely represent local adaptations to ecological features such as climate and habitat (Figure 1a, Figure S7). Rainforest sites have high annual rainfall and low inter‐annual variation in environmental variables, whereas the ecotone have lower annual rainfall and humidity, higher seasonality of rainfall and temperature, distinct predator communities and foods, and differences in ambient noise that influence greenbul song (Slabbekoorn & Smith, 2002; Smith et al., 2013). Montane habitats have lower minimum and mean annual temperatures, and vegetation communities that are distinct from lowland forest sites. These ecological differences have been shown to drive divergent selection resulting in genomic divergence among populations (Zhen et al., 2017).

Here, we first examine how the variation in environments inhabited by little greenbuls may have shaped genetic patterns under current climate conditions. We adopted the methodology and data from Zhen et al. (2017), summarized here. We used Restriction‐Site Associated (RAD)‐based sequences to analyze and compare genomes across 15 populations of greenbuls across four habitats in Central Africa (Figure 1a, two populations, in Gabon and Equatorial Guinea, were used in analyses but not shown in this figure). After identifying 47,482 SNPs with minor allele frequencies >2%, we determined the relationship between allele frequencies and current environmental variables (Figure 1b, Tables S3 and S4) using gradient forests (Ellis et al., 2012). Gradient forests have previously been used in ecology to assess turnover across species in a community (Roland Pitcher et al., 2012) as well as to model intraspecific molecular variation (Fitzpatrick & Keller, 2015). We found a total of 7238 SNPs that had a significant correlation with environmental variables (Zhen et al., 2017). The relationship between these SNPs and their (transformed) environmental correlates was reduced to two principal component axes and transformed into an RGB color scale to represent variation in the relationship between genomes and environments across populations (Figure 1b). We further visualize this relationship as “adaptive turnover,” a measure of how much the relationship between environment and genomics, as determined by gradient forest models, changes across the landscape. Adaptive turnover was estimated using focal statistics in ArcGIS (ESRI), where the range of RGB values around each pixel was calculated and visualized (Figure 1c). Higher ranges (red regions in Figure 1c) represent regions of high turnover in the relationship between genomes and environments, whereas low ranges (blue regions in Figure 1c) represent those regions that have more gradual transitions in this relationship across a landscape.

### 2.5 Estimating genomic changes required under future climate conditions

**FIGURE 2:**
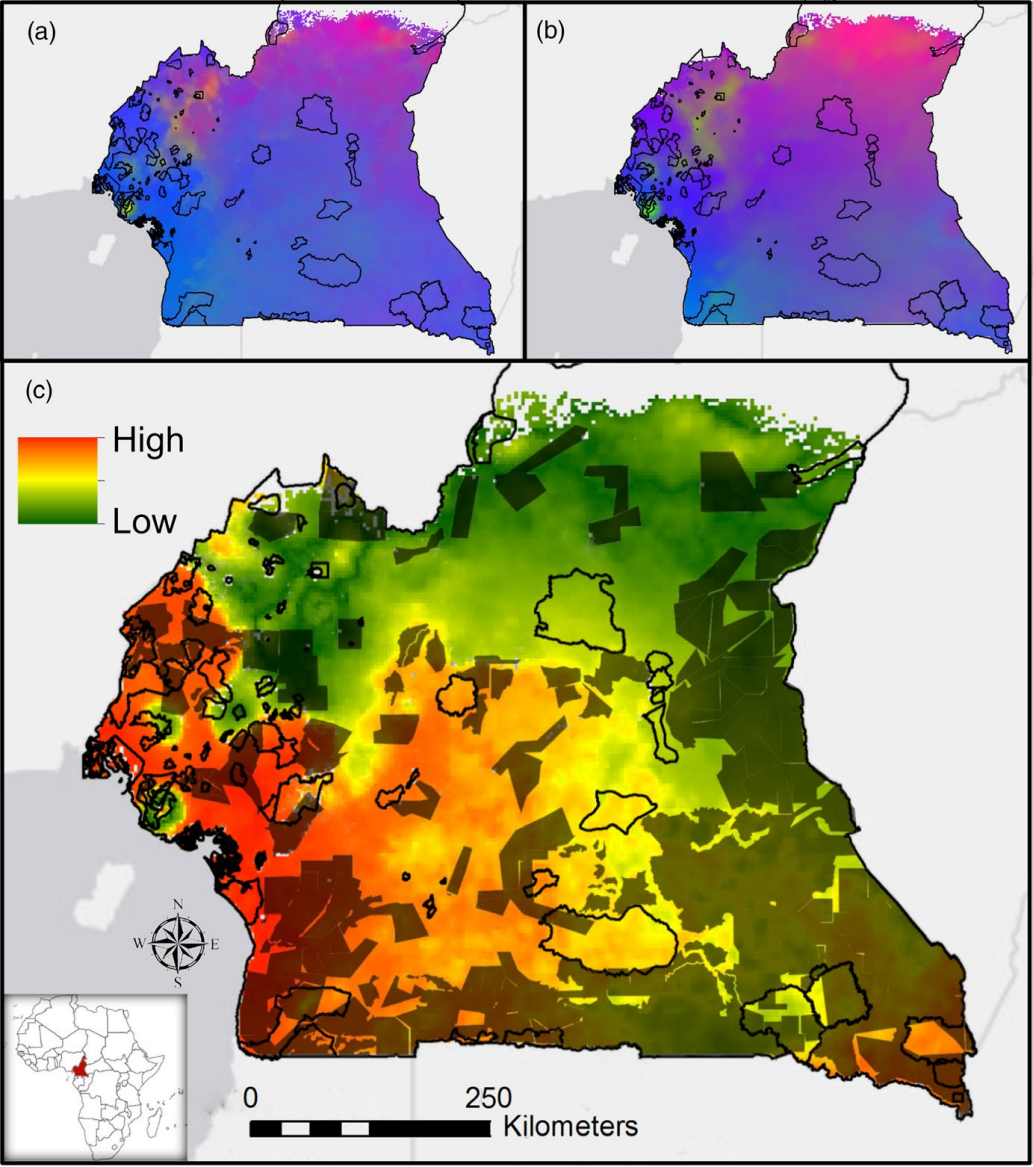
Patterns of genomic diversity and genomic vulnerability under current and future climate. (a) The relationship between current genomic and environmental variation of a species (same as Figure 1b). (b) The predicted genomic and environmental variation under future climate conditions (RCP 4.5 2080 scenario). (c) The absolute difference between (a) and (b) is the estimated genomic vulnerability under climate change. High vulnerability areas, shown in red, are where population genomes must change rapidly, and low vulnerability areas, shown in green, are where populations will need to change less to keep pace with climate change. Socio‐economic threats, indicated in black (includes logging and mining, see Figures S3–S5, S8, and S9 for additional threats) will limit conservation efforts in those areas. Ecotone regions, at the center part of the country, show relatively fewer current threats and low genomic vulnerability under climate change. In contrast, coastal and some southern regions show the highest genomic vulnerability and high socio‐economic threats. Current protected areas in the country are represented by black‐outlined polygons

In order to estimate how genomic–environmental associations of little greenbuls might change under future climate conditions, we projected the relationship under current conditions (from our model above, Figure 2a) onto the same variables under the IPCC Representative Concentration Pathway (RCP) 4.5 scenario for 2080 (Figure 2b) described by the IPCC as an intermediate scenario (Shukla et al., 2019). This procedure assumes that the current genome–environment relationships that have been shaped over the last 20,000 years will remain representative for the next 60 years. For example, if there is a linear relationship between alleles associated with traits important in temperature regulation in current populations of greenbuls, our approach assumes that the same relationship will persist over time and geographic space in 2080.

### 2.6 Assessing the mismatch between current and future genome–environment relationships

Based on the modeled genotype–environment relationships under current (Figure 2a) and future (Figure 2b) conditions, one can calculate the magnitude of mismatch between the two time periods, which represents the required genetic change for a population to adapt to the new environmental conditions. We thus compared the geographic distribution of environmentally associated intraspecific variation that has evolved over the last 20,000 years (current) to that projected under rapid climate change (future) and calculated the difference between the present and future genomic signatures as determined by our gradient forest models. We refer to this difference as *Genomic Vulnerability* (Bay et al., 2018). The calculation assumes that all alleles are equally important in their contribution to the overall relationship between genomes and environment, that these alleles are either under direct selection from the environment, or tightly linked to such regions, and that patterns of future geneflow do not change. For each grid cell (1 km^2^) across the range of the species, we visualized the Genomic Vulnerability (described above) as the dimensionless difference between current and future genomic signatures (Figure 2c).

## 3 RESULTS

### 3.1 Current and future socio‐economic impacts

The percent area covered by mining concessions in the Southwest region was significantly lower than in other regions of Cameroon (Figure S1; *t* = 2.087, df = 7, *p* = 0.0377, Cohen's *d* = 1.86). Not surprisingly, ecotonal regions have significantly lower levels of logging of any region of the country (Figure S2; test of percent logging concessions in ecotone vs. other regions: *t* = 1.891, df = 7, *p* = 0.05, Cohen's *d* = 1.77).

Predictions of future land use in 2080 suggest much of the greenbul's current habitat in rainforest, and ecotonal sites will be converted to agriculture and may no longer be available (Figure S8). The amount of suitable habitat will heavily depend on the type of agricultural system employed. For instance, strategies that include small woodlots and large hedgerows between fields could provide some viable habitat for greenbuls; however, it is unlikely that populations could be sustained if agriculture shifts toward large mechanized farms whose priority is to maximize land use. Our analysis suggests that due to land use changes, the largest greenbul populations assuming no change in traits associated with environment will be restricted to secondary forests and abandoned plantations of the ecotone and the gallery forests of Mbam Djerem National Parks, Mbéré Valley, and the forest on the northern slopes of Tchabal Mbabo and Tchabal Gandaba in the ecotone, a region currently under consideration as a National Park.

### 3.2 Current patterns of species richness, endemism, and adaptive turnover

Patterns of species richness and endemism and their relationships to threats from socio‐economic activities can be compared to our new measure of adaptive turnover (Figure 1c, Figure S10). Adaptive turnover was high in the ecotone between rainforest and savanna, in the northwest central portion of the country and along elevational gradients between lowlands and highlands in the north, central, and western portion of the country (Figure 1c). These regions have been identified previously as important areas for adaptive variation (Smith et al., 1997, 2014). Currently, fewer protected areas are located in the ecotone as compared to elsewhere in the country. Current protected areas harbor a higher proportion of adaptive turnover in the greenbul compared to its full range (Table S5, Figure 1c, Figure S10). Regions of high endemism also captured regions of higher average adaptive variation in the greenbul. This is primarily due to the northwestern highlands of Cameroon that rank high in both measures. Regions with high species richness, in contrast, had lower average adaptive turnover in the greenbul (Table S5, Figure 1c, Figure S10).

### 3.3 Future climate and genomic vulnerability

The minimum temperature of the coldest month was the most important variable for explaining the current spatial distribution of allele frequencies across the landscape (Table S1, Figure 2a). Under RCP 4.5, the minimum temperature is estimated to increase up to 2°C in coastal areas in 2080 (Figure S11). The implication is that there will be significant mismatches between current allele frequencies and those that will be required under a 2080 climate. As regions warm, directional selection may act on loci involved in temperature tolerance, favoring alleles that are adaptive for high temperatures.

#### 3.3.1 Genomic vulnerability

The mismatch between current and future genomic variation shows geographically distinct patterns (Figure 2c). High vulnerability areas shown in red are where population genomes are predicted to change rapidly, and low vulnerability areas shown in green are where populations are predicted to change less to keep pace with climate change. Socio‐economic threats and indicated in black (shown are major logging and mining concessions). These as well as potential other socio‐economic threats (Figures S3–S5, and S8) will limit conservation efforts in those areas. Ecotone regions, at the center part of the country, show relatively fewer current threats and low genomic vulnerability under climate change. In contrast, coastal and some southern regions show the highest genomic vulnerability and high socio‐economic threats. Results further suggest that in areas with the highest levels of Genomic Vulnerability, allele frequencies will need to change by as much as 100%, whereas at medium levels allele frequencies will need to change by 25–50%. Only in ecotonal and some northern montane regions will populations experience the lowest levels of Genomic Vulnerability, where allele frequencies are expected to change the least—no more than 25% of their current values (Figure S9).

## 4 DISCUSSION

Our results underscore the importance of adopting a comprehensive approach to conservation planning. Estimated vulnerability based solely on future climate change, while not considering other human impacts, will clearly limit conservation potential and outcomes. For example, while more northern areas of Cameroon clearly exhibit less Genomic Vulnerability, limited regions are predicted to not suffer from other anthropogenic pressures such as logging and mining.

Maximizing the adaptive potential of species in Central Africa will be an essential part of any global attempt to minimize extinctions. A previous phylogenetic analysis of 200 bird species, where the climatic niche was defined by temperature and precipitation, found that historical rates of temperature change have been ~1°C per million years (Quintero & Wiens, 2013). In stark contrast to this gradual change, seasonal warming in coastal Cameroon is predicted to exceed 2°C in the next 60 years.

With respect to the little greenbul, we predict that in some areas, changes in allele frequencies required to keep track with warming by 2080 would need to be of a similar magnitude to changes that have occurred since the LGM, approximately 18,000–20,000 years ago (Maley, 2001). However, in the most genomically vulnerable areas of Cameroon (bright red areas in Figure 2c), they will need to evolve at a rate faster than they have done since the LGM—a magnitude of change in 50 years likely beyond the limits of biological reality. Adaptation in areas with large anticipated temperature changes, such as coastal Cameroon, will require that standing genetic variation captures loci that are important for temperature tolerance in the future. In contrast, ecotone and some northern montane forest areas with high adaptive turnover (Figure 1c) and low Genomic Vulnerability (Figure 2c) may offer refugial areas for mitigating the impacts of climate change. Although these results only capture variation in a single species, they are exemplary for additional studies performed across multiple taxa from Central Africa that together can help inform a number of management approaches currently being proposed for preserving biodiversity in the face of rapid anthropogenic climate and land use change (reviewed in Moritz & Agudo, 2013). This type of approach could also be applied to identify climate refugia in the Congo Basin. While we focus on temperature because of the significant correlation between the minimum temperature of the coldest month and allele frequencies in the greenbul (Zhen et al., 2017), climatic variables such as precipitation may well be important for many other species.

It is important to emphasize that the present analyses implement the medium Representative Concentration Pathway (RCP) 4.5, according to which emissions increase until 2040, and then decrease. However, if one considers more extreme scenarios, such as RCP 8.5, in which emissions are assumed to continue to rise in the 21st century, the magnitude of warming will likely be considerably higher than 2°C. In fact, under RPC 8.5, mean annual temperature in eastern Cameroon is predicted to increase 3–7°C relative to the 1950–2000 baseline (Fuller et al., 2018). Moreover, the updated and improved Shared Socioeconomic Pathways (SSP) (O'Neill et al., 2017) scenarios of the Coupled Model Intercomparison Projects (CMIP) in the upcoming sixth IPCC Assessment Report suggest even more extreme warming under similar CO_2_ emissions (Riahi et al., 2017). While it is difficult to accurately predict the impacts of such high temperatures in a comprehensive fashion, such elevated mean temperatures and their associated extreme events might well push many species beyond their maximum critical temperatures leading to local extinctions and declines in ecosystem function (García et al., 2018).

When anthropogenic impacts other than climate warming are factored in, the fate of many populations becomes dire. Many regions already exhibit high rates of deforestation and fragmentation, which is likely to be exacerbated by large‐scale agriculture, making establishment of new protected areas less likely. Coastal and inland forest areas in southern Cameroon are already significantly constrained by existing land use, and those constraints are predicted to become more severe in the coming decades. The interior ecotone, in contrast, is predicted to experience smaller changes in land use and the minimum temperature of the coldest month than the coast and will likely represent one of a handful of areas in the country that will permit the persistence of populations. Currently, many Central African nations are experiencing rapid growth of urban human populations. However, under future climate change, the Cameroonian urban centers of Douala and Yaoundé are expected to experience extreme warming. This may lead to increasing urbanization and higher human population densities in the cooler, more northern regions of the country, putting further stress on these ecosystems (where genomic vulnerability is predicted to be lowest; Figure S9).

Using the association between the genome in the little greenbul and its environment under current and future climate conditions, we were able to estimate *Genomic Vulnerability* and reveal that coastal and central Cameroon populations will require the largest shifts in adaptive genomic variation. In contrast, in the more northern forest–savanna ecotones, genomic shifts were more moderate, and other anthropogenic impacts, such as mining and logging, were comparatively lower in magnitude. This suggests that if the goal is to preserve little greenbul populations and their adaptive potential, current forest–savanna ecotones are key. Of course, our analyses focused on only a single species, and data on diverse taxa (e.g., birds, mammals, insects and plants) will be necessary to develop comprehensive conservation strategies to mitigate various anthropogenic impacts and climate change. Below, we illustrate how our approach could be expanded from one species to many in order to better understand how communities of species may be affected.

**FIGURE 3:**
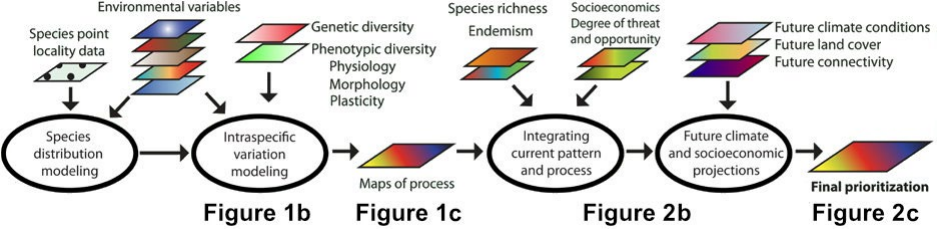
Framework to map current and potential future biodiversity across a landscape. The results, when combined with the degree of threat and socio‐economic impacts, can be used to prioritize areas of importance for conserving evolutionary processes under climate change (modified from Thomassen et al., 2011). Relevant figures are indicated at the bottom to illustrate respective steps that can be taken and combined for multiple species to build a comprehensive management strategy

The conceptual model for integrating the information in these analyses across multiple species and across a variety of input variables is shown in Figure 3 (modified from Thomassen et al., 2011). We present this as a possible exemplar for a framework to combine various types of data across species to generate a prioritization approach for a region. To clarify our single‐species example, relevant figures from our analyses here are shown along the bottom of Figure 3: (1) intraspecific genomic variation associated with environmental variables (Figure 1b); (2) levels of adaptive turnover, where regions with greater differences in genomic variation can be prioritized (Figure 1c); (3) integration of current pattern and process projected under future climate conditions (Figure 2b); (4) a map of areas of relative Genomic Vulnerability under a future climate scenario in conjunction with known socio‐economic threats (Figure 2c); and (5) a final prioritization map for all species (equivalent to Figure 2c, but compiled across multiple species is currently being pursued (Morgan et al., 2020).

Our results illustrate how evolutionary process may uniquely be combined with standard methods used in conservation planning and prioritization. It is often stated that to conserve biodiversity pattern and process, one has to map it, because conservation decision makers work with maps (M. Reynolds, Personal communications). Our future work aims to combine maps of adaptive turnover for a diverse set of taxonomic groups, including mammals, plants, insects, and amphibians, in conjunction with other components of phenotypic plasticity, behavioral traits, and demographic information that strengthen the approach. Ultimately, we believe qualitative comparisons between measures of biodiversity, land use patterns, and cultural acceptance of protected areas can allow stakeholders to make quantitative comparisons and examine trade‐offs to make the results both more robust and actionable for conservation planning.

It is no longer adequate to base conservation decisions on current distributions of species, yet our results highlight the challenges of conservation planning in a rapidly changing anthropocentric world. The integrated conceptual framework presented in Figure 3 suggests that considering intraspecific variation in extant populations and projecting how variation may be distributed under future climate, in the context of other anthropogenic changes, will be essential. In the case of the little greenbul, the changes will likely be dramatic.

## CONFLICT OF INTEREST

None declared.

## Supporting information

Supplementary Material

## Data Availability

All of the genetic data collected for this study are available in public databases (see Zhen et al., 2017): RADseq data—NCBI SRA database BioProject ID PRJNA390986; RNAseq data—NCBI SRA database BioProject ID PRJNA390772; Data files including RAD loci consensus sequences, VCF file and sample information available at Dryad doi: https://doi.org/10.5061/dryad.8n8t0.
