# Supplementary material for: Genomic vulnerability and socio‐economic threats under climate change in an African rainforest bird
Source: Evol Appl. 2021 Jan 28;14(5):1239–47. doi: 10.1111/eva.13193 (PMC8127712; doi:10.1111/eva.13193)
Supplement: Supplementary file 1 — Supplementary Material [file EVA-14-1239-s001.docx]

**Supplemental Materials**

We considered sites as conservation targets based on current intraspecific genetic variation (defined as areas of “adaptive turnover”, represented in Fig. 1c). Regions showing high adaptive turnover were classified as higher conservation priority for the little greenbul. We carried out separate analyses identifying sites with high species richness (amphibians, birds, and mammals) and high abundance of endemic species (amphibians, birds, freshwater fish, mammals, plants, and reptiles) (Table S5). We then compared these traditional conservation metrics of endemism and richness, and current protected areas with our calculated adaptive turnover in greenbuls (Fig. 1c, represented by a dimensionless change in color value on an RGB color scale, as in Bay *et al*. 2018) using calculated summary statistics: 1) across the range of greenbuls in Cameroon, 2) within currently protected areas, 3) within regions of high species richness, and 4) within regions of high species endemism (Fig. S10, Table S5). We then compared our results involving biological processes with socio-economic pressures. We assessed the overlap between adaptive turnover and current threats from human population density, logging, mining, major agriculture, proposed dams and rail, and roads (Figs. S1-S5).

In addition to considering present anthropogenic threats, it is also crucial to prioritize regions based on future impacts of climate change. Based on genotype-environment relationships modeled across contemporary populations, one can calculate the amount of mismatch between current genomic variation and future variation required for a population to adapt to environmental change. As noted in the main text, we refer to this mismatch as genomic vulnerability (Bay et al. 2018). To estimate genomic vulnerability, we projected the present genome-environment relationships onto the predicted future climate conditions. After estimating genomic vulnerability under climate change (Fig. 2c), we carried out a separate analysis of threats from land use change using the IMAGE land cover model (Table S1).


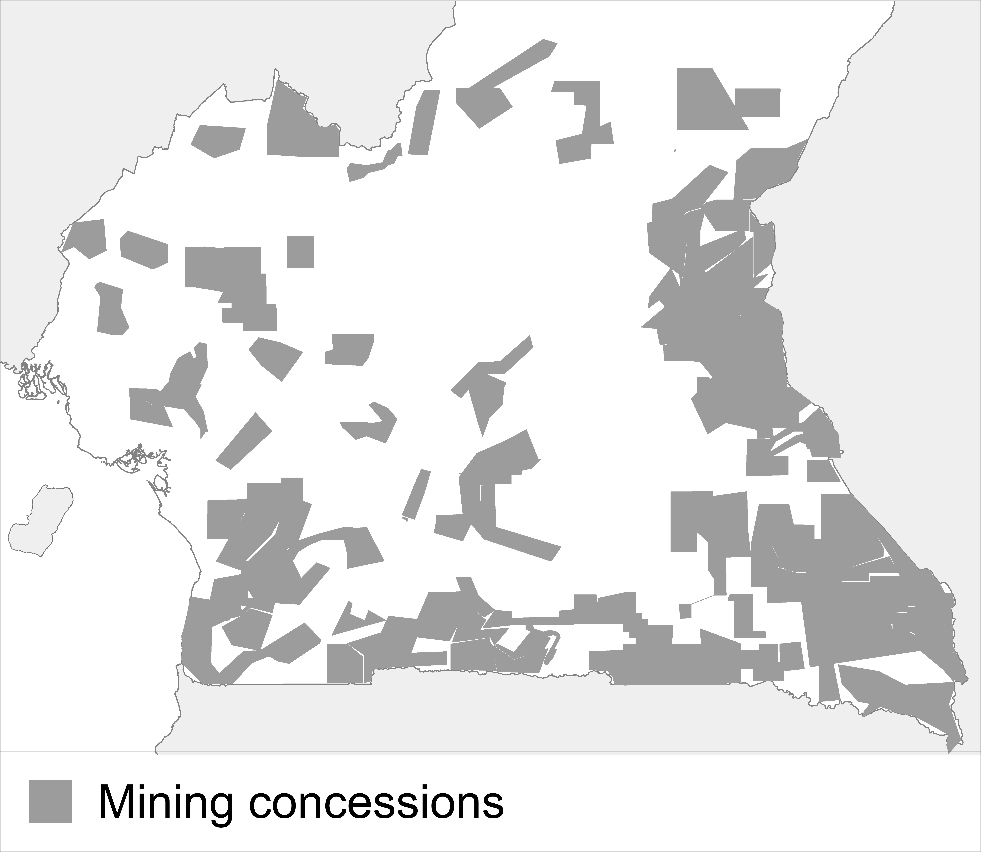


**Figure S1**

Mining concessions, as reported from Cameroon’s Ministry of Mine and Technological Development and the World Resources Institute (World Resources Institute and Cameroon Ministry of Mines and Technological Development 2016).


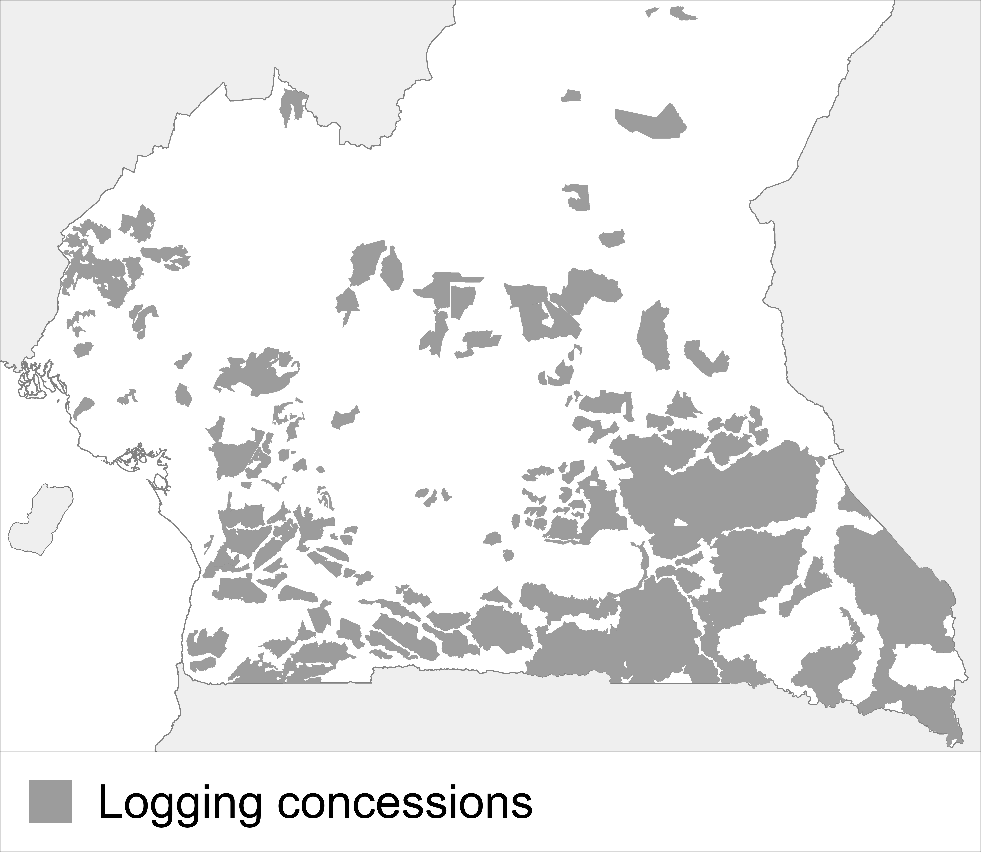


**Figure S2**

Logging concessions, as reported by Cameroon’s Ministry of Forestry and Wildlife and the World Resources Institute (WRI 2012). With respect to logging concessions, in each region of Cameroon, we calculated: (1) active cutting areas where timber harvest is ongoing, and (2) production forests where timber harvest is permitted by law but is not taking place currently. The ecotone has no active cutting areas, and the number of production forests is lower than any other region of Cameroon within the greenbul’s range.


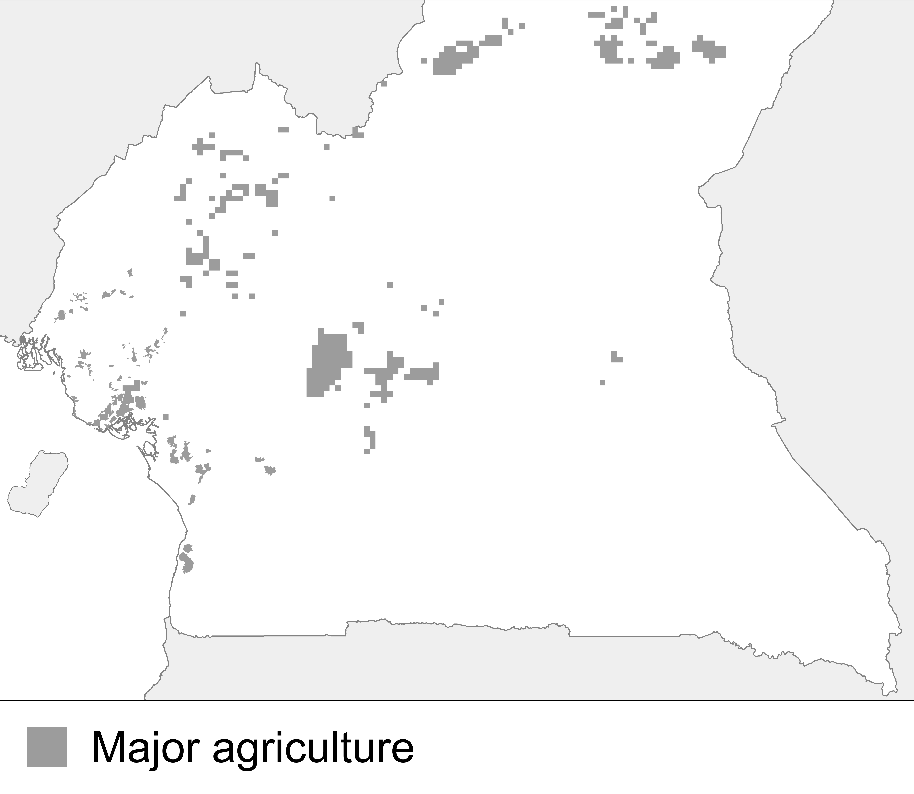


**Figure S3**

Major agriculture activity in regions of Cameroon (present-day).


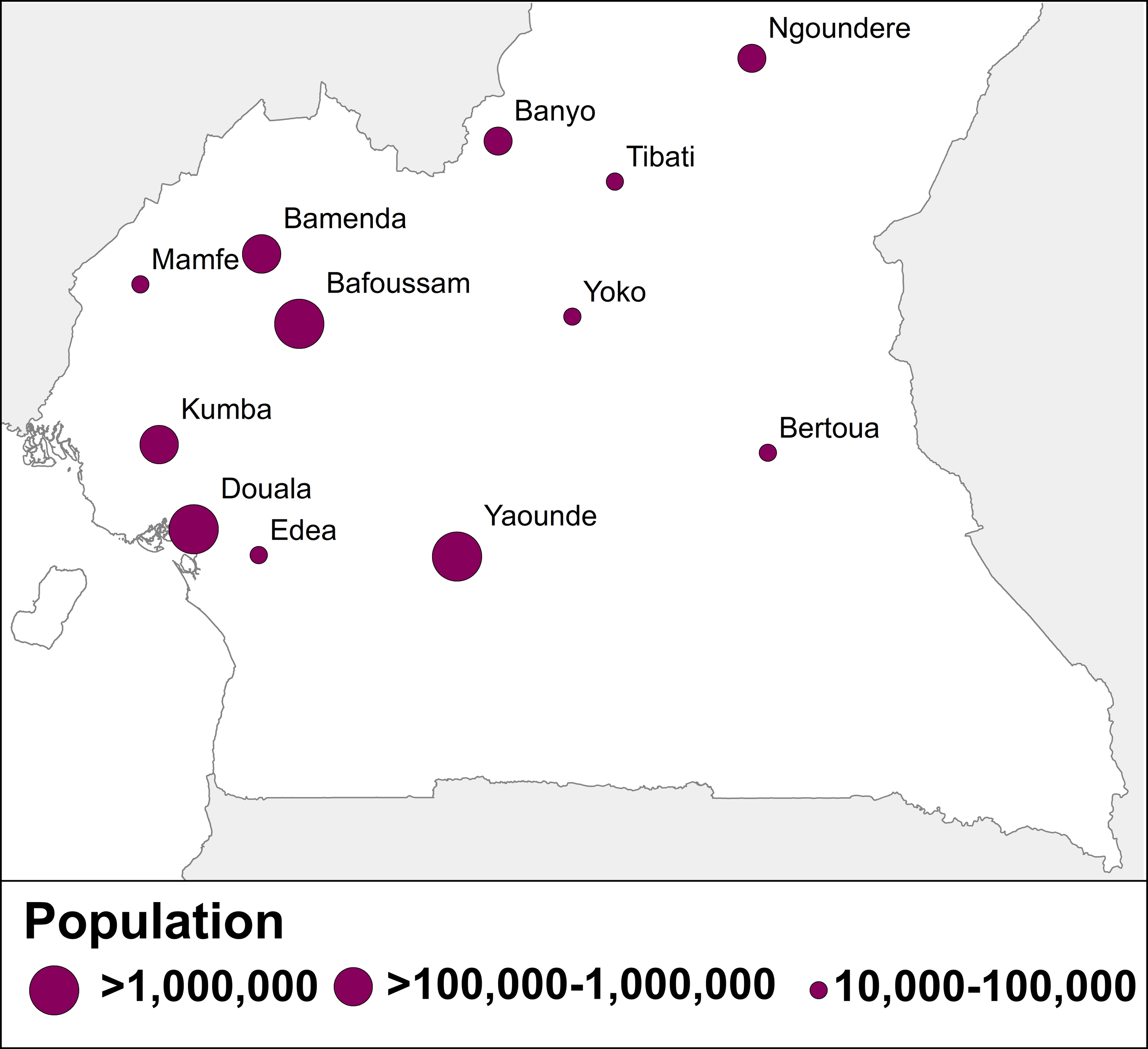


**Figure S4**

Present day human populations of major towns and cities.


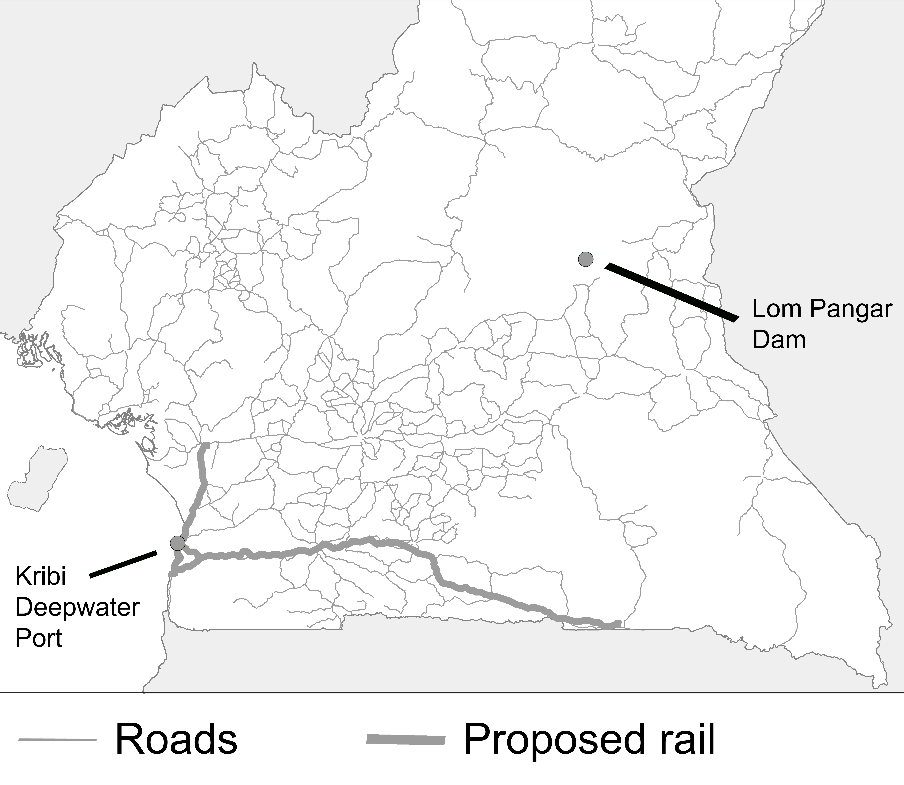


**Figure S5**

Major infrastructure and transportation projects (present and future activities).


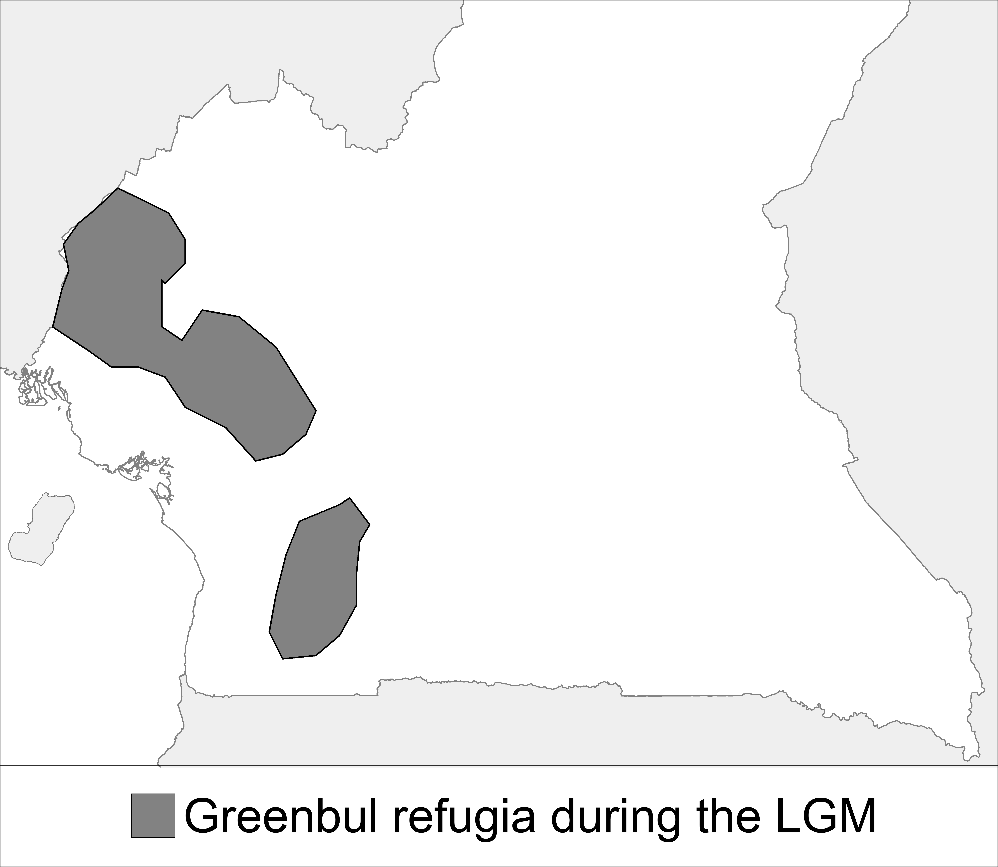


**Figure S6**

Hypothesized refugia during the LGM (Maley 2001; Dalibard et al. 2014).


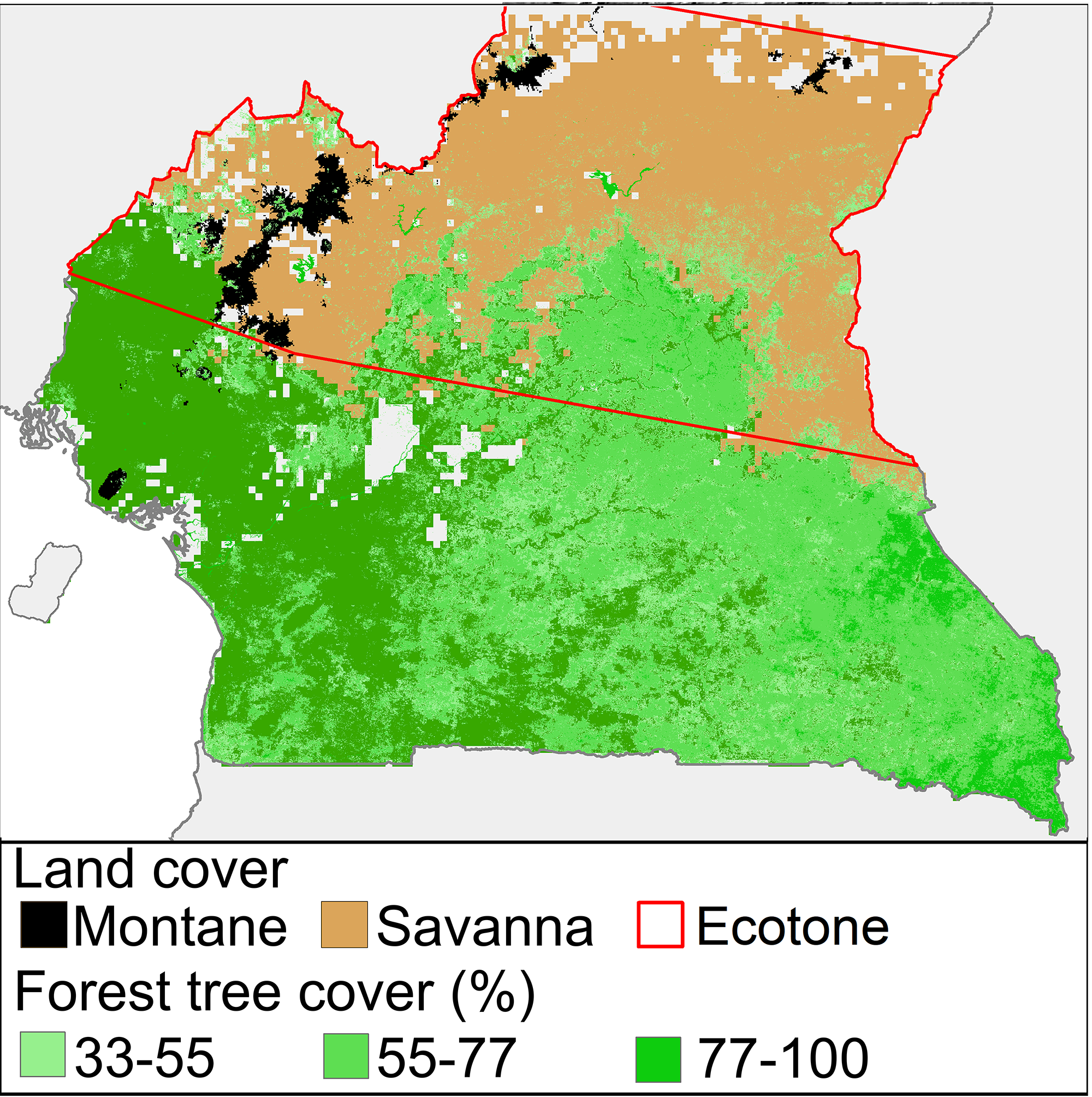


**Figure S7**

Land cover of Cameroon. The ecotone was defined based on environmental characteristics, such as percent tree cover, as described elsewhere (Slabbekoorn and Smith 2002; Smith et al. 2013).

Gray represents habitat unsuitable for the Greenbul such as zones of commercial agriculture (Fig. S3) and cities (Fig. S4).


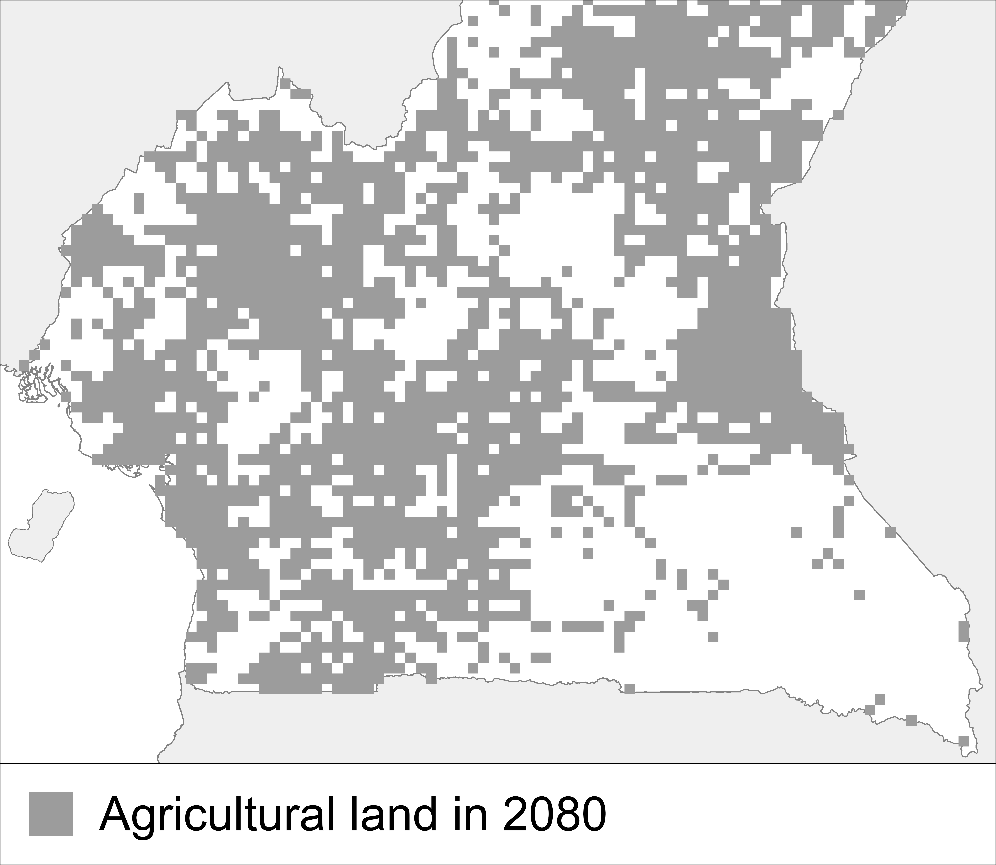


**Figure S8**

Projected land conversion to agriculture under RCP 4.5 in 2080.


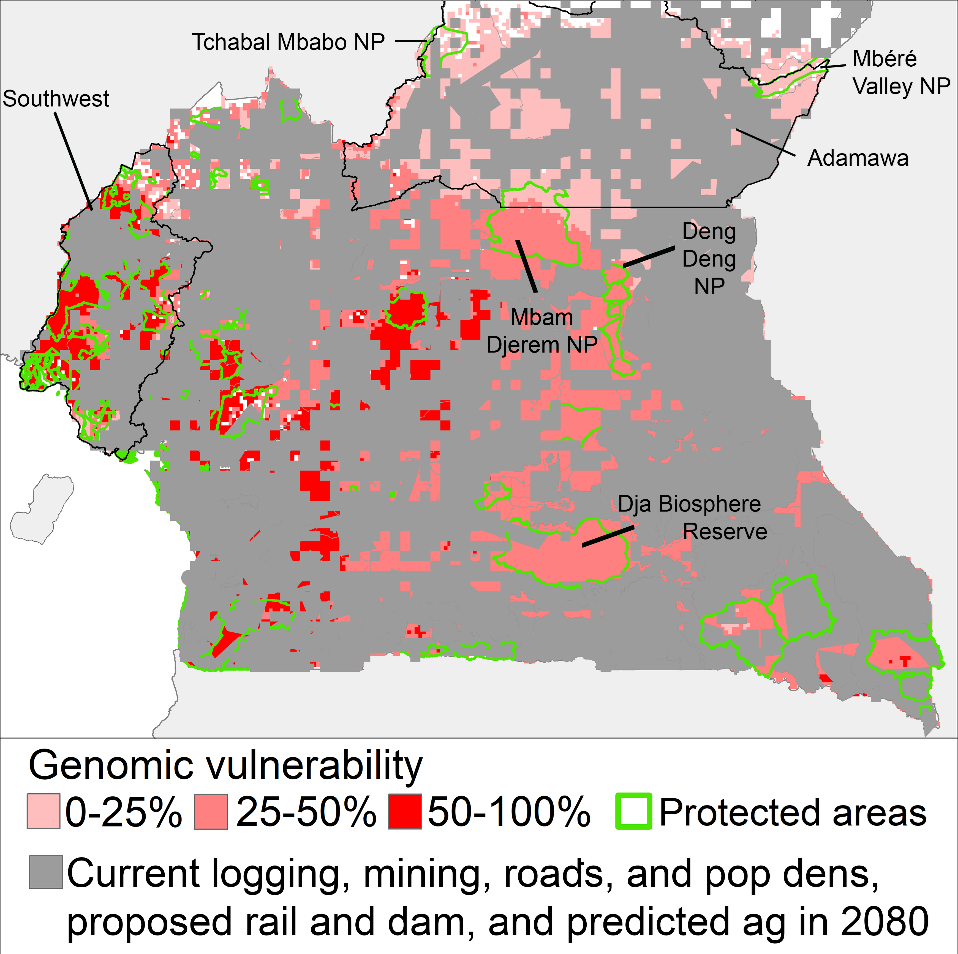


**Figure S9**

Predicted land use in 2080 and the spatial distribution of vulnerability to climate change. According to the IMAGE model, by 2080 much of the region will be threatened by development. NP = national parks. Ag = agriculture. “Pop dens” denotes regions with at least 250 inhabitants per km^2^.


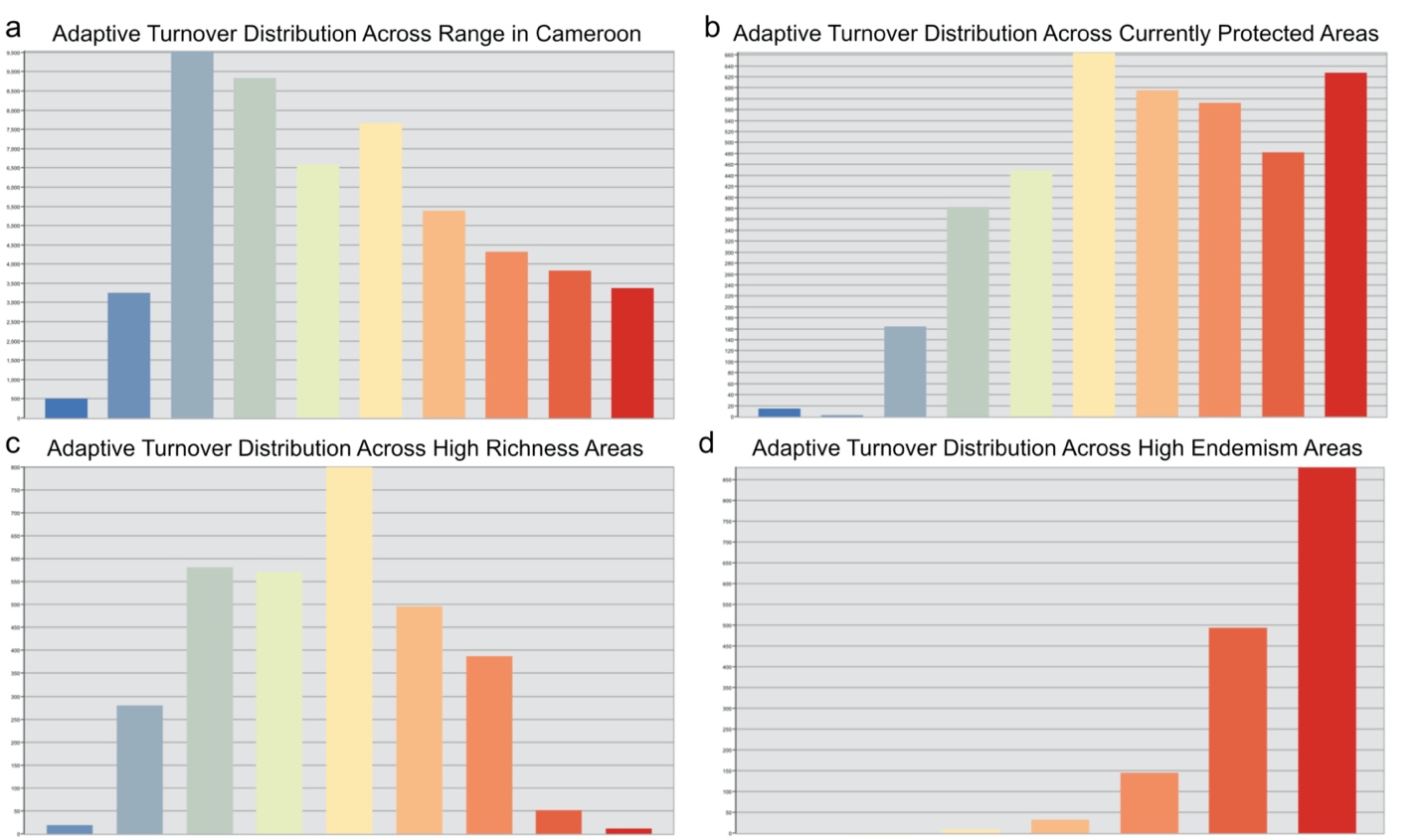


**Figure S10**

Distribution of adaptive turnover within: a) the full range of the little greenbul in Cameroon, b) current protected areas, c) regions of high species richness, and d) regions of high species endemism. Currently protected areas (b) and regions of high species endemism (d) skew towards capturing higher amounts of adaptive turnover than the full distribution (a), whereas regions of high species richness (c) skews towards capturing lower amounts of adaptive turnover. Colors of scalebars are identical for each panel, and correspond to the same values as those presented in Fig. 1c. For each pixel in the stated range, values are the dimensionless standard deviation of the eight surrounding pixels, ranging from 0-255 based on RGB values calculated from the results of the gradient forest model between genomes and environments (Fig. 1b). Larger values indicate greater standard deviations around a pixel, and represent a larger change in the relationship between genomes/environment across a given distance.


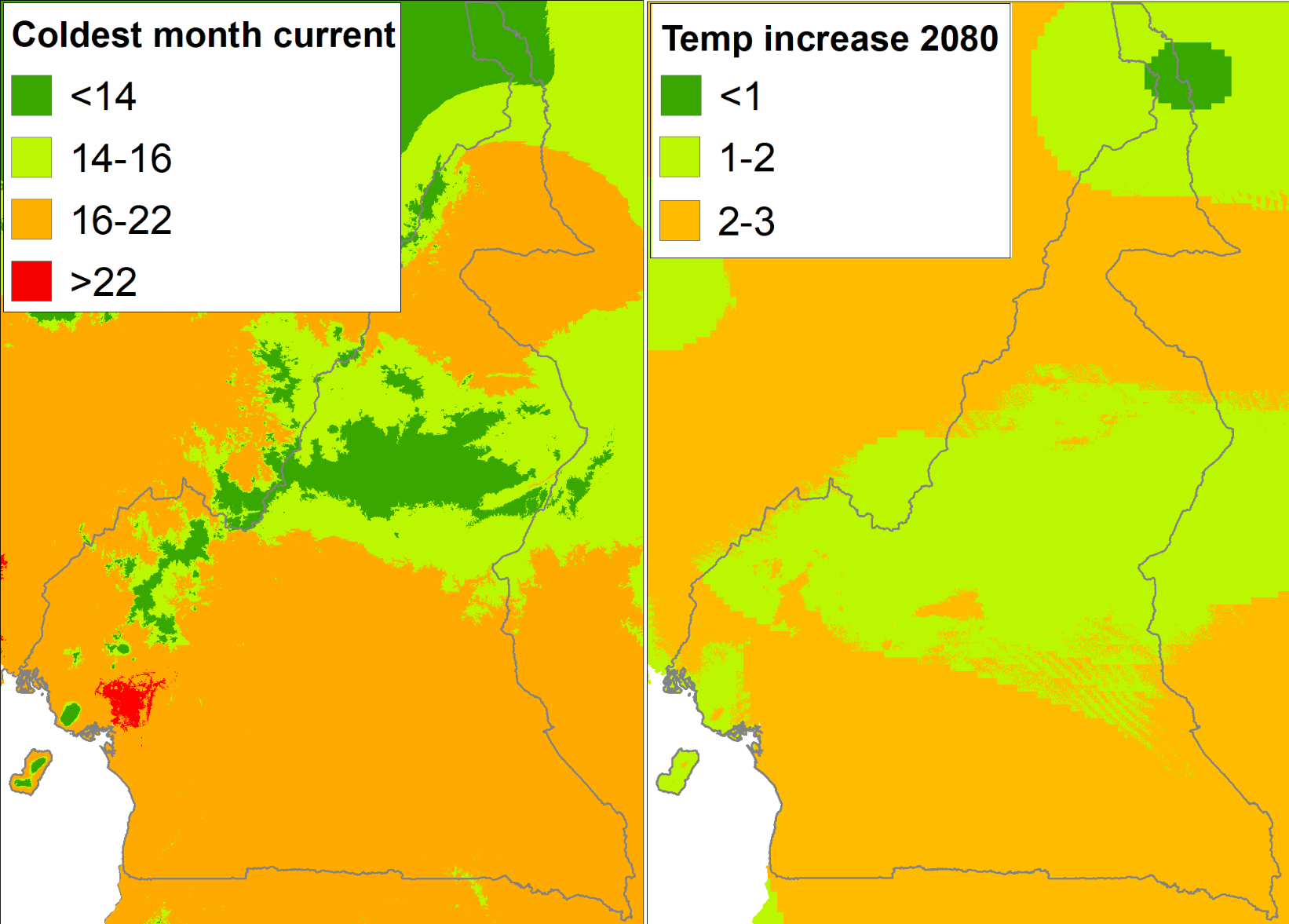


**Fig. S11**

Minimum temperature of the coldest month under current conditions (left) and the temperature increase under RCP 4.5 in 2080 (right).

**Table S1 | Current and late 21^st^ Century anthropogenic threats. With respect to human population density, we assume 250 or more inhabitants per km^2^ reduces greenbul habitat suitability.**

| **Threat** | **Year** | **Ref. and Notes** |
| --- | --- | --- |
| Logging concessions | 2016 | (WRI 2012) updated in 2016 |
| Mining concessions | 2013 | (World Resources Institute and Cameroon Ministry of Mines and Technological Development 2016) |
| Large scale agriculture | 2012 | (Friedl et al. 2002) updated in 2012 |
| Roads | 2013 | (WRI 2012) updated in 2013 |
| Population density | 2016 | (Dobson et al. 2000) updated in 2016 |
| Proposed railroads | Slated for construction by 2020 | (Ministère de l’Economie de la Planification et de l’Aménagement du Territoire 2012) |
| Lom Pongar dam | Currently under construction | (Mbonteh 2017) |
| Kribi Deepwater Port | Currently under construction | (Dominguez-Torres and Foster 2011; Tumanjong 2015) |
| Land conversion to agriculture in 2080 under RCP 4.5 | Predicted using IMAGE 3.0 model | (van Vuuren et al. 2017) |

**Table S2 | Traditional measures of biodiversity that we compared with adaptive turnover.** Richness and endemism are two of the most widely utilized criteria for identifying areas of high conservation value (Meir et al. 2004; Ceballos 2007). The richness and endemism data were global in scale. We limited the spatial scale of the analysis to the ecological niche of the greenbul in Cameroon as defined by Smith *et al.* (Smith et al. 2013).

| **Measure** | **Taxa** | **Ref.** |
| --- | --- | --- |
| Richness | Amphibians, birds, and mammals | (Jenkins et al. 2013) |
| Endemism | Amphibians, birds, freshwater fish, mammals, plants, and reptiles | (Hoekstra et al. 2010) |

**Table S3 | Climatic variables used to construct the spatial model of greenbul population structure in Zhen *et al*. (2017) (see Fig. 1b).** The climatic data was constructed via interpolation from a 50-year time series of weather station records with a thin-plate spline that incorporates elevation, latitude, and longitude as covariates (Hijmans et al. 2005; Fick and Hijmans 2017). The weather stations include such sources as the Global Historical Climatology Network (GHCN), the United Nations Food and Agricultural Organization (FAO), the World Meteorological Organization (WMO), the International Center for Tropical Agriculture (CIAT), and additional country-based station networks (Buermann et al. 2008).

| **Variable** | **Units** | **Min** | **Max** |
| --- | --- | --- | --- |
| Minimum temperature of the coldest month | °C | -1.1 | 23.1 |
| Mean diurnal range [mean of monthly (max temp – min temp)] | °C | 6.4 | 15.3 |
| Annual mean temperature | °C | 4.7 | 28.5 |
| Precipitation of the driest quarter | mm | 0 | 548 |
| Total annual precipitation | mm | 318 | 4,206 |
| Precipitation seasonality (coefficient of variation) | dimensionless | 37 | 158 |
| Precipitation of the wettest quarter | mm | 275 | 1892 |
| Temperature seasonality (standard deviation × 100) | dimensionless | 4.6 | 31.8 |
| Maximum temperature of the warmest month | °C | 11 | 41 |

**Table S4 | Vegetation and landscape variables used to construct the spatial model of greenbul population structure in Zhen *et al.* (2017). (see Fig. 1b)** We used eight variables from satellite sensors that measure the properties of forest vegetation including canopy closure, canopy roughness, deciduousness, greenness, and normalized difference vegetation index (Long et al. 2001; Hansen et al. 2002; Myneni et al. 2002; Huete et al. 2006; Thomassen et al. 2011). Previous work has demonstrated a high correlation between ground-based measurements of forest properties and these remotely sensed variables (Saatchi et al. 2007; Buermann et al. 2008). Our vegetation data were constructed from satellite images in publicly available databases: NASA Earthdata Search (https://search.earthdata.nasa.gov/search) and the Scatterometer Climate Record Pathfinder (<http://www.scp.byu.edu/>).

| **Variable** | **Remote sensing (RS) sensor** | **Vegetation/landscape parameter** | **RS metrics** | **Ref.** |
| --- | --- | --- | --- | --- |
| Digital elevation (derived from the original 90 m resolution) | SRTM | Surface elevation | SRTM-HGT-M: mean elevation STRM-HGT-S: standard deviation | (Farr et al. 2007) |
| Scatterometer backscatter | QuikSCAT | Canopy roughness, leaf/wood density, and vegetation deciduousness | QSCAT-M: mean backscatter  QSCAT-S: standard deviation of backscatter | (Long et al. 2001) |
| Percent tree cover | MODIS | Forest cover, heterogeneity | VCF: continuous field product | (Tucker et al. 2005) |
| Normalized Difference Vegetation Index (NDVI) | MODIS | Vegetation type and seasonality | NDVI-1: maximum NDVI  NDVI-2: mean NDVI  NDVI-3: mean NDVI of green band | (Hansen et al. 2002) |

**Table S5 | Summary statistics of adaptive turnover in the little greenbul across by characteristic of regions.** Higher mean values of adaptive turnover in currently protected park areas and regions of high endemism suggest that these areas are capturing more intraspecific variation related to environmental variables per square area than is observed in either the full range of the species across Cameroon, or in regions identified as containing high species richness.

| **Adaptive Turnover** | Endemism | Richness | Protected | Full Range |
| --- | --- | --- | --- | --- |
| **Minimum** | 0.43 | 0.09 | 0 | 0 |
| **Maximum** | 20.01 | 6.3 | 18.93 | 20.03 |
| **Mean** | 4.75 | 0.78 | 2.04 | 1.64 |
| **Standard Deviation** | 2.56 | 0.56 | 2.37 | 1.95 |

References

Bay, R. A., R. J. Harrigan, C. L. Underwood, H. L. Gibbs, T. B. Smith, and K. C. Ruegg. 2018. Genomic signals of selection predict climate-driven population declines. *Science* 359 (6371):83-6.

Buermann, W., S. Saatchi, T. B. Smith, B. R. Zutta, J. A. Chaves, B. Mila, and C. H. Graham. 2008. Predicting species distributions across the Amazonian and Andean regions using remote sensing data. *Journal of Biogeography* 35 (7):1160-1176.

Ceballos, G. 2007. Conservation priorities for mammals in megadiverse Mexico: The efficiency of reserve networks. *Ecological Applications* 17 (2):569-578.

Dalibard, M., S. M. Popescu, J. Maley, F. Baudin, M. C. Melinte-Dobrinescu, B. Pittet, T. Marsset, B. Dennielou, L. Droz, and J. P. Suc. 2014. High-resolution vegetation history of West Africa during the last 145 ka. *Geobios* 47 (4):183-198.

Dobson, J. E., E. A. Bright, P. R. Coleman, R. C. Durfee, and B. A. Worley. 2000. LandScan: A global population database for estimating populations at risk. *Photogrammetric Engineering and Remote Sensing* 66 (7):849-857.

Dominguez-Torres, C. and V. Foster. 2011. *Cameroon's Infrastructure: A Continental Perspective. Africa Infrastructure Country Diagnostic*. Washington, DC: World Bank.

Farr, T. G., P. A. Rosen, E. Caro, R. Crippen, R. Duren, S. Hensley, M. Kobrick, M. Paller, E. Rodriguez, L. Roth, D. Seal, S. Shaffer, J. Shimada, J. Umland, M. Werner, M. Oskin, D. Burbank, and D. Alsdorf. 2007. The Shuttle Radar Topography Mission. *Reviews of Geophysics* 45 (2):RG2004.

Fick, S. E. and R. J. Hijmans. 2017. Worldclim 2: New 1-km spatial resolution climate surfaces for global land areas. *International Journal of Climatology* 37 (12):4302-4315.

Friedl, M. A., D. K. McIver, J. C. F. Hodges, X. Y. Zhang, D. Muchoney, A. H. Strahler, C. E. Woodcock, S. Gopal, A. Schneider, A. Cooper, A. Baccini, F. Gao, and C. Schaaf. 2002. Global land cover mapping from MODIS: algorithms and early results. *Remote Sensing of Environment* 83 (1-2):287-302.

Hansen, M. C., R. S. DeFries, J. R. G. Townshend, R. Sohlberg, C. Dimiceli, and M. Carroll. 2002. Towards an operational MODIS continuous field of percent tree cover algorithm: examples using AVHRR and MODIS data. *Remote Sensing of Environment* 83 (1-2):303-319.

Hijmans, R. J., S. E. Cameron, J. L. Parra, P. G. Jones, and A. Jarvis. 2005. Very high resolution interpolated climate surfaces for global land areas. *International Journal of Climatology* 25 (15):1965-1978.

Hoekstra, J., J. L. Molnar, M. Jennings, C. Revenga, M. D. Spalding, T. M. Bouver, J. C. Robertson, T. J. Heibel, and K. Ellison. 2010. *The Atlas of Global Conservation. Changes, Challenges, and Opportunities to Make a Difference*. Oakland: University of California Pres.

Huete, A. R., K. Didan, Y. E. Shimabukuro, P. Ratana, S. R. Saleska, L. R. Hutyra, W. Z. Yang, R. R. Nemani, and R. Myneni. 2006. Amazon rainforests green-up with sunlight in dry season. *Geophysical Research Letters* 33 (6).

Jenkins, C. N., S. L. Pimm, and L. N. Joppa. 2013. Global patterns of terrestrial vertebrate diversity and conservation. *Proceedings of the National Academy of Sciences* 110 (28):E2602-E2610.

Long, D. G., M. R. Drinkwater, B. Holt, S. Saatchi, and C. Bertoia. 2001. Global ice and land climate studies using scatterometer image data. *EOS, Transactions of the American Geophysical Union* 82 (43):503.

Maley, J. 2001. The impact of arid phases on the African rain forest through geological history. In *African Rain Forest Ecology and Conservation: An Interdisciplinary Perspective*, edited by W. Weber, L. White, A. Vedder and L. Naughton-Treves. New Haven, Connecticut: Yale University Press.

Mbonteh, R. 2017. Cameroon: Lom-Pangar Dam - Construction of Power Plant Launched. *allAfrica*.

Meir, E., S. Andelman, and H. P. Possingham. 2004. Does conservation planning matter in a dynamic and uncertain world? *Ecology Letters* 7 (8):615-622.

Ministère de l’Economie de la Planification et de l’Aménagement du Territoire. 2012. *Le plan directeur ferroviaire national du Cameroun*. Yaounde: Ministère de l’Economie, de la Planification et de l’Aménagement du Territoire.

Myneni, R. B., S. Hoffman, Y. Knyazikhin, J. L. Privette, J. Glassy, Y. Tian, Y. Wang, X. Song, Y. Zhang, G. R. Smith, A. Lotsch, M. Friedl, J. T. Morisette, P. Votava, R. R. Nemani, and S. W. Running. 2002. Global products of vegetation leaf area and fraction absorbed PAR from year one of MODIS data. *Remote Sensing of Environment* 83 (1-2):214-231.

Saatchi, S., R.A. Houghton, R.C. dos Santos Alvalá, J.V. Soares, and Y. Yu. 2007. Distribution of aboveground live biomass in the Amazon basin. *Global Change Biology* 13:816-837.

Slabbekoorn, H. and T. B. Smith. 2002. Habitat-dependent song divergence in the Little Greenbul: an analysis of environmental selection pressures on acoustic signals *Evolution* 56 (9):1849-1858.

Smith, T. B., R. J. Harrigan, A. N. G. Kirschel, W. Buermann, S. Saatchi, D. T. Blumstein, S. R. de Kort, and H. Slabbekoorn. 2013. Predicting bird song from space. *Evolutionary Applications* 6 (6):865-874.

Thomassen, H. A., T. Fuller, W. Buermann, B. Mila, C. M. Kieswetter, P. Jarrin, S. E. Cameron, E. Mason, R. Schweizer, J. Schlunegger, J. Chan, O. Wang, M. Peralvo, C. J. Schneider, C. H. Graham, J. P. Pollinger, S. Saatchi, R. K. Wayne, and T. B. Smith. 2011. Mapping evolutionary process: a multi-taxa approach to conservation prioritization. *Evolutionary Applications* 4 (2):397-413.

Tucker, C. J., J. E. Pinzon, M. E. Brown, D. A. Slayback, E. W. Pak, R. Mahoney, E. F. Vermote, and N. El Saleous. 2005. An extended AVHRR 8-km NDVI dataset compatible with MODIS and SPOT vegetation NDVI data. *International Journal of Remote Sensing* 26 (20):4485-4498.

Tumanjong, E. 2015. Cameroon chooses Sino-French conglomerate for crucial Kribi Port contract. 28 August 2015. *Dow Jones Newswire*.

van Vuuren, D. P., E. Stehfest, Dehj Gernaat, J. C. Doelman, M. Van den Berg, M. Harmsen, H. S. de Boer, L. F. Bouwman, V. Daioglou, O. Y. Edelenbosch, B. Girod, T. Kram, L. Lassaletta, P. L. Lucas, H. van Meijl, C. Muller, B. J. van Ruijven, S. van der Sluis, and A. Tabeau. 2017. Energy, land-use and greenhouse gas emissions trajectories under a green growth paradigm. *Global Environmental Change-Human and Policy Dimensions* 42:237-250.

World Resources Institute and Cameroon Ministry of Mines and Technological Development. 2016. *Cameroon Mining Permits. On-line: http://gis-gfw.wri.org/arcgis/rest/services/country_data/africa/MapServer/2*. Washington, DC and Yaounde, Cameroon: World Resources Institute and Cameroon Ministry of Mines and Technological Development,.

WRI. 2012. *Interactive Forest Atlas of Cameroon. Version 3.0*. Washington, DC: World Resources Institute.

Zhen, Y., R. J. Harrigan, K. Ruegg, E. C. Anderson, T. C. Ng, S. Lao, K. E. Lohmueller, and T. B. Smith. 2017. Genomic divergence across ecological gradients in a Central African rainforest songbird (*Andropadus virens*). *Molecular Ecology* 26:4966-77.
